# A Manual of Nursing in Pelvic Surgery

**Published:** 1894-08

**Authors:** 


A Manual of Nursing in Pelvic Surgery. By L. S. McMurtry, A.M., M.D. John P. Morton & Co., Louisville, Publishers, 1894.

A little hand-book prepared by the author originally for the use of the nurses under his personal direction. After a brief consideration of the location of the pelvic organ of the female, directions are given for the preparation of intruments and patient; the duties of the nurse during and after operation; complications liable to follow, etc. A useful pocket manual for both hospital and private nurses.

